# Woman at Home

**Published:** 1887-11-19

**Authors:** 


### The Doctor's Pulpit.

### WOMAN AT HOME.

Our plain words on this topic have been so kindly received, particularly by women, that we are encouraged to continue with the view of entering more into detail. To make the subject practical it will be well to do as the old-fashioned clergy used to do, and consider it under several heads. The particular point in this week's discourse shall be "The Home and Its Furnishing." If anyone says "What has the doctor to do with that?" We answer, "Everything."

The mere mention of the home brings such a variety of affairs to the mind, all of them important from a health point of view, that the temptation is to condense them into the form of question and answer, and construct a "Home Catechism." The first question in such a catechism would naturally be—What is a home? Is it a house? And if it is a house, is it a house alone, or a house plus a thousand additional circumstances which merely to write down would be to use up all our available space? Happily, there is no need to define. Those who have homes know what they are by their possession, and those who have not know perhaps what they are by memory; or, in the case of anyone who never had homes at all, imagination may very well take the place of knowledge.

A home presupposes a house as its primary condition, not lodgings. Unhappy are those young people who begin their married life in lodgings. Every properly-mated pair of birds should have a proper nest—a nest of their own providing. The home may be large or small, a flat, as in Scotland, or a whole house as in England. For young people there are certain advantages in the "flat" system; and as the occupant of "The Doctor's Pulpit" is not a Scotchman, he may be allowed the claim to speak without partiality and without prejudice. For small families, where few bed-rooms are required, a flat will give larger rooms for the same or even a smaller rent than a whole house. By living in flats also a better neighbourhood may be selected without the incurring of additional expense. In cases, therefore, where the income at first is moderate, a flat is well worth a consideration.

This question of flat or whole house especially concerns the young wife. For if, as will often happen, she commences life with one, or at most two servants, not only will she find a house of one storey and fewer rooms more easily kept clean, but the absence of stairs will be discovered to be a great saving of servant power. But, whether flat or otherwise, a house must be had, and we shall take it for granted that it will be of such a rent as can be paid without difficulty. That should be considered imperative. Not that we think Love always flies out at the window when Poverty comes in at the door—far from it. But we are persuaded that the entrance of Poverty at the door makes Love at least look at the window and wish to fly out; and in those not infrequent cases where Poverty plainly indicates that he has come "for a permanency," Love will sometimes make a frantic dash for the window and fly away like Noah's dove on her third departure, to return again no more.

But why contemplate such a miserable possibility? Our young housewife, who is to be duly *trained* beforehand, will see that a house is chosen of such a rent as shall bear a due proportion to her husband's secure income. And what proportion is that? A tenth, our grandfathers used to say; we have got down to a seventh or an eighth. It is impossible to say exactly, so much depends upon circumstances. But in cases where the house is not also the place of business, as it is with the young doctor, it will be well that the rent should not be more than from a tenth to a seventh of the whole income.

The choice of house and furniture will naturally be one of the first ventures in the new co-partnery. The firm of husband and wife should discuss the subject with all the serious wisdom which the greatness of the occasion demands. The medical preacher suggests that it would be well at the outset to decide exactly how much can be spent on furniture, and on no account to go beyond the sum agreed upon. A house may be like a pocket with a hole in it: however much you put in, it is never full. A line, therefore, should be drawn somewhere. A particular friend of the writer's decided in this way, and when he and his young wife came to the end of the fixed sum it was found that no mangle had been bought. "Very well," said Benedict, "then we will draw the line at mangles;" and at mangles the line was accordingly drawn. It may be encouraging to add that in due time that very necessary article was also bought and paid for.

What are the three principal things to be desired in a home? Are they not these—health, comfort, elegance? Health, the doctor naturally and properly places first; and being an Englishman, he as naturally puts comfort second, and elegance last. Not that he thinks elegance unimportant, but health he regards as essential, and comfort as the *sine qua non* of health. For health the rooms must be of a fairly good size, and the drainage and other sanitary arrangements perfect. Is it the wife's duty to see to this—is it not rather the husband's? It is the duty of both the members of the firm, as much that of the wife as of the husband. And indeed the wife will have to spend many more hours daily in the house than the husband, and she will have infinitely more opportunities of finding out defects of sanitation than he will. We hold firmly that every woman who enters upon the duties of house management should know all about cisterns, drains, sinks, water pipes, kitchen boilers, and every other department of domestic sanitation. Some people may say, This is absurd! We answer, Those are absurd who say so. It is absolutely essential in these days of large towns, and knavish builders, that some member of the family understand and attend to the domestic sanitation. And how can the husband be expected to do this, who generally leaves home as soon as he has swallowed his breakfast in the morning, and does not return again until the close of the day? This much is certain, that if a man's wife really loves him as she should, she will consider it her duty to save him from every domestic worry she possibly can; and this is one of those worries, at any rate in London, which a practical woman can very well manage to save her husband from. Of course, if the lady be one of those superlative creatures who marry in order that they may have a home and position without working for them, she will think these ideas ridiculous and unpractical. As we cannot hope to convince a person like her we must leave her to enjoy her own opinions. Everyone admits that it is a wife's duty to have her house clean and pretty. But in whatever degree health is of greater importance than comfort and pleasure, in that degree should the mistress of the house consider it her duty to have it healthy as well as clean and pretty. Volumes might be written on this topic; but we think that every right-minded woman will at once appreciate the force of the argument.

Comfort is not, according to some people, a very exalted or poetical idea; but it is eminently practical. Comfortable and enjoyable meals, a pleasant room, and a very easy chair to recline in after the fatigues and worries of the day; a nice fresh bed-room, with clean and wholesome bedding; a good fire by day and night when the weather is cold and cheerless—These are the little lubricators which oil the wheels of the domestic machine and make it run smoothly, pleasantly, and without wear and tear. All these the mistress of the house can secure for the family; all these she longs to secure, as a rule; but she often fails from want of training. Mothers and daughters, and fathers too, should recognise that all these things are included in woman's specialty; and as she is a specialist of such pre-eminent importance to the world, they should think it one of their chief duties towards her to have her thoroughly prepared for this great and pre-eminent special department in the general economy of life.

